# Maintenance of patient-reported health-related quality of life post neoadjuvant relugolix prior to the initiation of prostate radiation therapy

**DOI:** 10.3389/fonc.2024.1496646

**Published:** 2025-01-17

**Authors:** Min Jung Koh, Min Ji Koh, Jessica Y. Hsueh, Lindsey Gallagher, Malika Danner, Alan Zwart, Marilyn Ayoob, Deepak Kumar, Michael Carrasquilla, Paul Leger, Nancy A. Dawson, Simeng Suy, Sean P. Collins

**Affiliations:** ^1^ Department of Radiation Medicine, MedStar Georgetown University Hospital, Washington, DC, United States; ^2^ Department of Therapeutic Radiology, Yale University School of Medicine, New Haven, CT, United States; ^3^ University of South Florida (USF) Health Morsani College of Medicine, Tampa, FL, United States; ^4^ Biotechnology Research Institute, North Carolina Central University, Durham, NC, United States; ^5^ Department of Oncology, Lombardi Comprehensive Cancer Center, Georgetown University Medical Center, Washington, DC, United States

**Keywords:** prostate cancer, androgen deprivation therapy, SBRT, health related quality of life, EQ-5D

## Abstract

**Introduction:**

Studies have demonstrated that injectable GnRH receptor agonists further suppress cancer progression when paired with radiotherapy (RT) in patients with intermediate- to high-risk prostate adenocarcinoma. Relugolix is a newly available oral GnRH receptor antagonist that achieves swift and profound castration (total testosterone <20 ng/dl) at high rates, which may shape patients’ health-related quality of life. The main objective of this prospective study was to explore the effects of neoadjuvant relugolix on health-related quality of life in prostate cancer patients immediately prior to stereotactic body radiation therapy (SBRT).

**Methods:**

Patients treated at Georgetown between January 2021 and September 2023 with neoadjuvant relugolix per an institutional protocol were included in the study (IRB 12-1775). The five-item EQ-5D-3L, a well-established tool for quantifying patient-reported health status, was administered to each patient at baseline (prior to relugolix treatment) and again 1 h before the start of SBRT. Higher EQ Visual Analog Scale (VAS) overall scores reflected better quality of life (range 0 to 100). In line with the questionnaire framework, individual elements (mobility, self-care, usual activities, pain/discomfort, and anxiety/depression) were rated on a three-point scale from 1 (no problems) to 3 (severe problems). McNemar’s test and paired-sample t-test were performed to analyze changes pre- and post-relugolix treatment. Our investigation determined clinical significance based on minimally important difference (MID) calculated as 0.5 times the baseline standard deviation.

**Results:**

Among the 87 patients, average age was 71 years, 42% were non-white, and 24% were considered obese (BMI ≥30 kg/m²). Relugolix was initiated a median of 4 months before SBRT initiation (IQR: 3.9–5.4), with 87% of patients reaching profound castration (<20 ng/dl). The VAS overall score was notably higher at baseline (mean ± SD: 82 ± 10) compared to the paired score before RT (79 ± 14, p = 0.02), although this difference was not clinically significant. No statistically or clinically significant changes were observed in any of the five individual items.

**Conclusion:**

The use of neoadjuvant relugolix prior to prostate radiation therapy had no clinically significant impact on patient-reported health-related quality of life. Moreover, no statistically significant reductions were observed in any of the five individual health-related quality of life measures. As a key direction for future research, relugolix-associated changes to healthy-related quality of life should be contrasted to those brought about by injectable GnRH agonists.

## 1 Introduction

Prostate cancer continues to be one of the most prevalent malignancies affecting men worldwide, with localized cases often requiring a combination of therapeutic approaches to optimize outcomes. The National Comprehensive Cancer Network (NCCN) guidelines suggest that patients with unfavorable localized prostate cancer should undergo a combination of radiation therapy (RT) and androgen deprivation therapy (ADT) (1). ADT paired with conventionally fractionated RT substantially enhances metastases-free and overall survival (2). Emerging data also indicate that adding ADT to ultrahypofractionated RT, or stereotactic body radiation therapy (SBRT), for unfavorable prostate cancer may lower local disease persistence and biochemical recurrence comparable to SBRT alone (3, 4). Despite its effectiveness, ADT in combination with RT continues to be underutilized likely due to concerns over its bothersome side effects and the associated risks of exacerbating cardiovascular comorbidities (5, 6).

In 2020, the FDA granted approval for relugolix, an oral antagonist of gonadotropin-releasing hormone (GnRH) receptors. By directly inhibiting the release of luteinizing hormone (LH) and follicle-stimulating hormone (FSH), relugolix induces a swift reduction of testosterone levels to significant castration levels (total testosterone 0.7 nmol/L, <20 ng/dl) (7). The phase 3 HERO trial (NCT03085095) compared the effectiveness of this oral GnRH receptor antagonist versus GnRH agonist leuprolide and demonstrated that relugolix outperformed leuprolide in achieving and maintaining castration (total testosterone 1.73 nmol/L, <50 ng/dl) (8). By day 29 of treatment, 95% of patients on relugolix attained profound castrate levels compared to just 57% of those receiving leuprolide (8). Among patients taking relugolix, 96.7% maintained castration after 48 weeks versus 88.8% of patients on leuprolide (8). Notably, there were no statistical differences in rates of hormonal toxicities such as generalized fatigue, hot flashes, and musculoskeletal pain (8).

Neoadjuvant/adjuvant relugolix administered over 6 months has been examined in unfavorable localized prostate cancer when alongside conventionally fractionated RT (79.2 Gy in 44 fractions) (9). This combination resulted in 95% achieving castration and 87% reaching profound castration (9). However, the high incidence of rapid profound castration may influence health-related quality of life, particularly among minority and underserved populations, as these groups have previously been reported to exhibit greater rates of non-adherence to hormonal therapies (10). Furthermore, patient-specific characteristics, such as age or comorbidities, may amplify these effects. We conducted a prospective study to evaluate the influence of neoadjuvant relugolix on health-related quality of life (HRQoL) in intermediate- and high-risk prostate cancer patients prior to starting SBRT.

## 2 Materials and methods

Our team carried out a prospective study of subjects with unfavorable, localized prostate adenocarcinoma treated at Georgetown University Hospital (IRB 12-1175). As per institutional protocol, patients received a short-term treatment of relugolix for 6 months, along with SBRT. We reviewed patients’ medical records to gather demographic and oncologic information, including age, ethnicity, body mass index (BMI), prostate volume, pretreatment PSA levels, T stage, Gleason score, and treatment dosage. D’Amico criteria were utilized to categorize patient risk groups.

### 2.1 Pharmacologic treatment

Relugolix was started no less than 2 months before SBRT, beginning with a 360-mg loading dose on day 1, followed by an oral dose of 120 mg daily.

### 2.2 Follow-up and assessment

Total testosterone levels were measured concurrently with questionnaire administration. Serum testosterone under 50 ng/dl (<1.73 nmol/L) defined effective castration, while levels below 20 ng/dl (<0.7 nmol/L) defined profound castration (8). Each patient’s health status was assessed using the validated five-item EQ-5D-3L questionnaire collected at baseline (before initiating relugolix) and again 1 h prior to SBRT (11). The EQ VAS score spans from 0 to 100, with 0 reflecting the worst conceivable health and 100 reflecting the best conceivable health (11). The minimally important difference (MID) in the EQ VAS score was specified as a change of one-half standard deviation (SD) from the baseline (12). Individual items, including mobility, self-care, usual activities, pain/discomfort, and anxiety/depression, were scored on a three-point scale: 1 (no problems), 2 (some problems), and 3 (extreme problems) (11). For example, a patient who reports no problems in all five dimensions of EQ-5D-3L is described to have a health state of “11111” (11).

### 2.3 Statistical methods

Continuous variables were summarized by mean and standard deviation when normally distributed and compared using t-test. Non-normally distributed, continuous variables were calculated based on median and interquartile range and compared between groups with Wilcoxon rank-sum test. All categorical variables were summarized by frequencies and percentages and were compared using Pearson’s Chi-squared test and Fisher’s exact test. We assessed changes before and after relugolix treatment with McNemar’s test and paired-sample t-test for categorical and continuous variables, respectively. A p-value <0.05 determined statistical significance. Clinical significance was evaluated based on MID. All analyses were performed using R version 4.3.2 or a more recent version, as provided by The R Foundation of Statistical Computing (http://www.r-project.org/).

## 3 Results

### 3.1 Baseline demographic, clinical, and treatment characteristics

**Table 1** outlines the demographic, treatment, and tumor characteristics. From January 2021 to September 2023, 87 patients with intermediate- and high-risk prostate cancer were treated at Georgetown University Hospital. Patient ages ranged from 49 to 87 years, with an average age of 71. Most patients identified as Caucasian (57%) or African American (32%). A significant portion of the participants were considered overweight (48%) or obese (24%) with a BMI of 25–29.9 and ≥30 kg/m², respectively. Before treatment, the median PSA was 8.2 ng/ml, with levels ranging from 2.3 to 40.0 ng/ml. Most patients (71%) were ≥Grade Group 3. In terms of risk stratification, 69 patients (79%) had intermediate-risk disease and 17 (20%) were considered high-risk. The majority of patients had none (54%) to mild (34%) Charlson Comorbidity Index. The median time from relugolix initiation to SBRT was 4 months (IQR: 3.9–5.4 months). By SBRT initiation, 95% of patients had achieved effective castration (testosterone ≤50 ng/dl), and 87% had reached profound castration (testosterone ≤20 ng/dl).

**Table 1:** Demographic and clinical characteristics.

Characteristics	No. (%)			p-Value^a^
	All (n = 87)	EQ VAS overall Diff <0 (n = 30)	EQ VAS overall Diff ≥ 0 (n = 49)	
Age at baseline (y), mean ± SD	71 ± 8	71 ± 9	70 ± 7	0.9
<60	7 (8)	2 (6)	4 (8)	0.7^b^
60–69	33 (38)	11 (37)	20 (41)	
70–79	37 (43)	12 (40)	21 (43)	
>80	10 (11)	5 (17)	4 (8)	
Race				
White	50 (58)	17 (57)	29 (59)	0.8^b^
Black	28 (32)	9 (30)	16 (33)	
Other	9 (10)	4 (13)	4 (8)	
Gleason score				
3 + 3 = 6	5 (6)	2 (7)	2 (4)	0.9^b^
3 + 4 = 7	20 (23)	6 (20)	13 (27)	
4 + 3 = 7	47 (54)	18 (60)	25 (51)	
4 + 4 = 8	14 (16)	4 (13)	8 (16)	
4 + 5 = 9	1 (1)	0	1 (2)	
Risk group				
Low	0	0	0	0.9^b^
Intermediate	69 (79)	25 (83)	38 (78)	
High	17 (20)	5 (17)	10 (20)	
Recurrent	1 (1)	0	1 (2)	
Charlson Comorbidity Index, median (IQR)	0 (0–1)	0 (0–1)	0 (0–1)	0.1
None	47 (54)	16 (53)	27 (55)	0.6^b^
Mild (1–2)	30 (34)	12 (40)	16 (33)	
Moderate (3–4)	6 (7)	2 (7)	3 (6)	
Severe (>5)	4 (5)	0	3 (6)	
BMI (kg/m^2^), median (IQR)	27 (25–30) (n = 82)	26 (24–29)	27 (25–29) (n = 45)	0.3
Underweight (<18.5)	0	0	0	0.056^b^
Healthy weight (18.5 ≤ n < 25)	23 (28)	12 (40)	7 (16)	
Overweight (25 ≤ n < 30)	39 (48)	11 (37)	27 (60)	
Obese (≥30)	20 (24)	7 (23)	11 (24)	
Unknown	5	0	4	
Prostate volume (cc), median (IQR)	37 (28–50) (n = 86)	36 (27–45) (n = 29)	37 (29–50)	0.7
PSA (ng/ml), median (IQR)				
Baseline	8.2 (5.9–11.6)	8.3 (5.9–11.4)	8.2 (5.8–10.9)	0.6
At RT	0.5 (0.2–1.5) (n = 72)	0.6 (0.3–1.2) (n = 25)	0.4 (0.15–1.3) (n = 39)	0.2
At Start of SBRT				
Effective castration (testosterone <50 ng/dl)				
Yes	83 (95)	29 (97)	46 (94)	>0.99^b^
No	4 (5)	1 (3)	3 (6)	
Profound castration (testosterone <20 ng/dl)				
Yes	76 (87)	26 (87)	43 (88)	>0.99^b^
No	11 (13)	4 (13)	6 (12)	

a p-Values for the comparison between patients with EQ VAS Overall score difference (start–baseline) less than 0 versus greater or equal to 0 were calculated using t-test, Wilcoxon rank sum test, and chi-square test for normally distributed continuous, non-normally distributed continuous, and categorical variables, respectively.

b p-Values based on Fisher’s exact test due to some small cell counts.

### 3.2 Problems reported on the EQ-5D-3L dimensions and EQ VAS score

**Figure 1:**
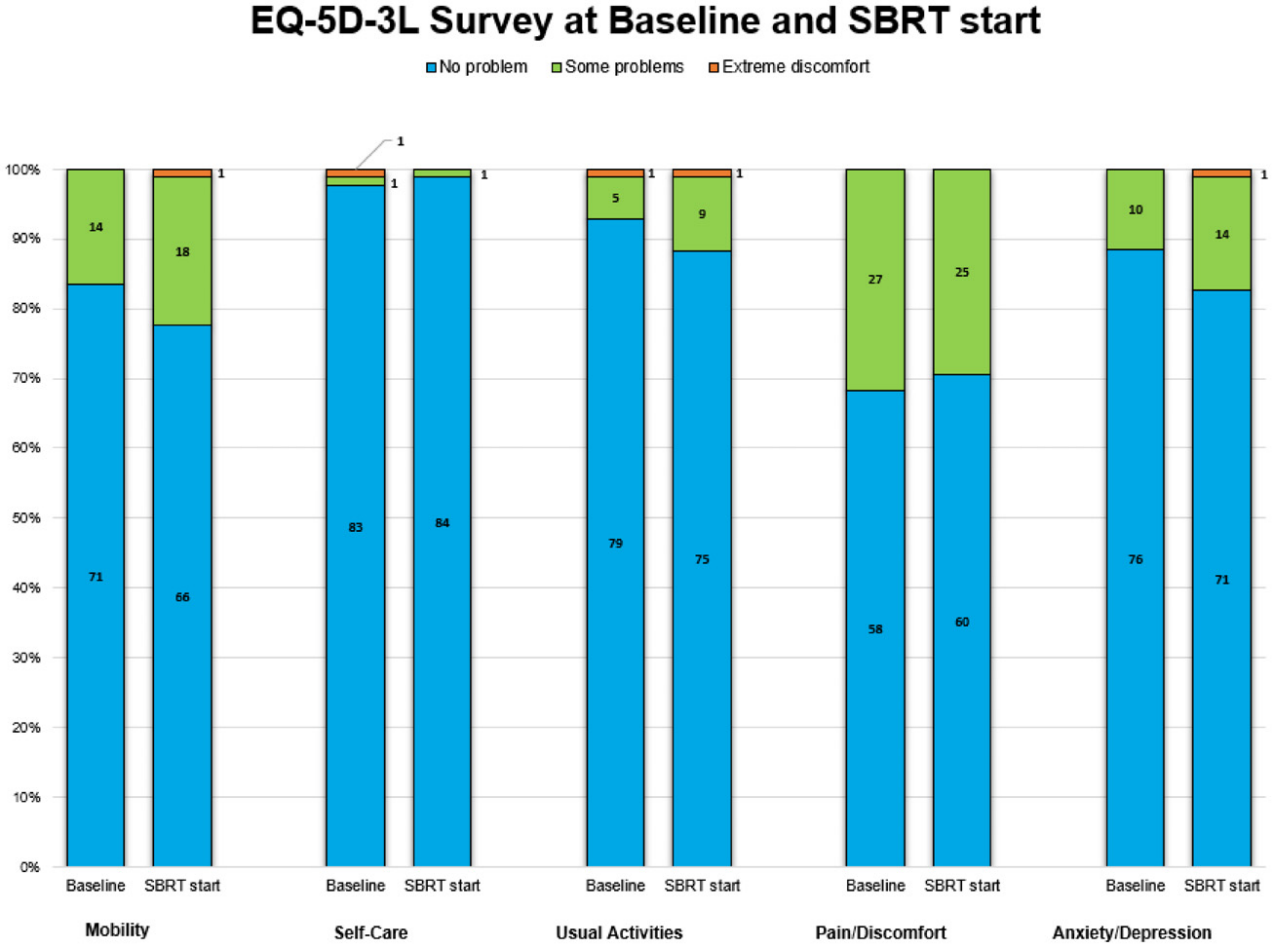
Proportions of three discomfort levels in each of five dimensions in stacked bar plots. The number in black on the bar plots denotes the number of patients.

Patient-reported health status outcomes measured by EQ-5D-3L and EQ-VAS score are reported in **Table 2**. A total of 79 patients completed the VAS questionnaire, while 85 completed the EQ-5D-3L. Baseline health-related problems were relatively uncommon in our patients; the majority (56%) of them had a good health state, defined as “11111.” Prior to treatment, the pain/discomfort dimension (32%) had the highest proportion of reported problems (some or extreme), followed by mobility (16%), anxiety/depression (12%), usual activities (7%), and self-care (2%) (**Figure 1**).

**Table 2:** EQ VAS, EQ-5D-3L baseline and start of RT (post hormonal therapy).

Characteristics	No. (%)		p-Value^a^	Clinical significance
	Baseline	Start		
VAS score overall, mean ± SD	82 ± 10 (n = 79)	79 ± 14 (n = 79)	0.02	NS
5D sections^#^ Mobility Self-care Usual activities Pain/discomfort Anxiety/depression	71/85 (84) 83/85 (98) 79/85 (93) 58/85 (68) 76/86 (88)	66/85 (78) 84/85 (99) 75/85 (88) 60/85 (71) 71/86 (83)	0.2 >0.99 0.4 0.8 0.2	NS NS NS NS NS
Full health (“11111”)	48/85 (56)	49/85 (58)	>0.99	NS

^#^No. (%) of patients with no problems (point = 1) in each section.

a p-Values comparing two time points were calculated using the McNemar’s test and paired-sample t-test for categorical and continuous variables, respectively.

NS, non-significant.

**Figure 2:**
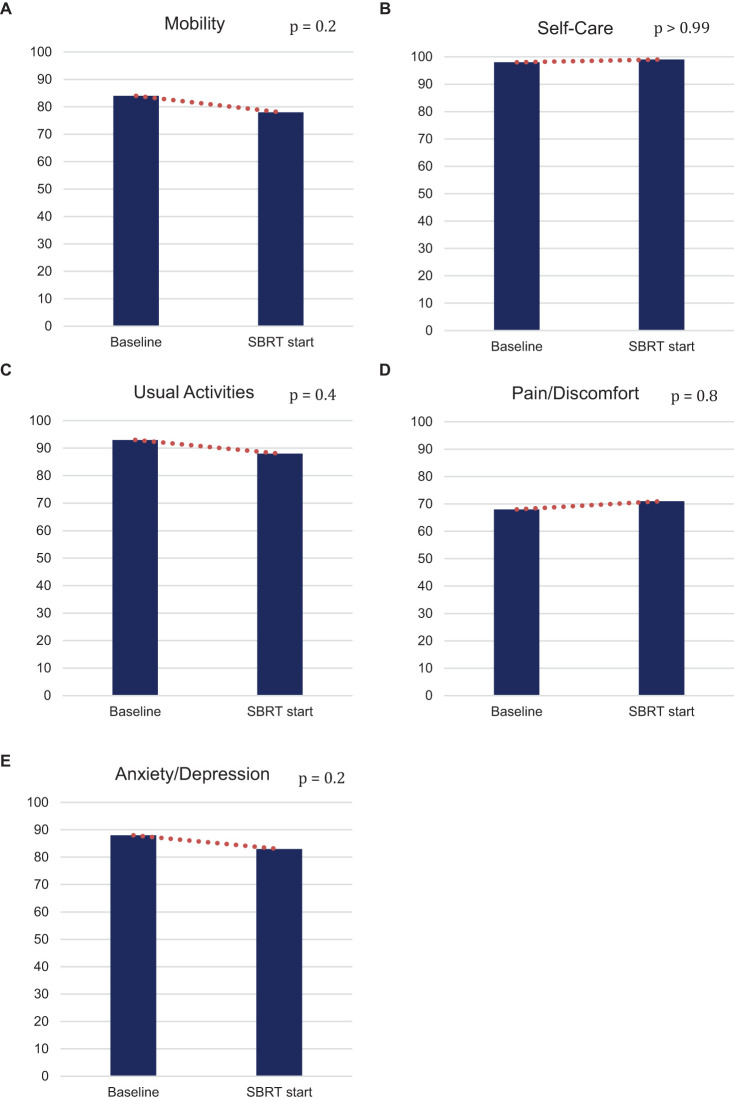
Percentage of patients who do not have any problem (EQ-5D-3L) in **(A)** mobility, **(B)** self-care, **(C)** usual activities, **(D)** pain/discomfort, and **(E)** anxiety/depression at baseline and at SBRT initiation.

Post hormonal therapy, a slightly higher number (58%) of patients were in good health state “11111.” The pain/discomfort dimension (29%) remained the highest proportion of five problems reported. The order remained the same as baseline: mobility (22%), anxiety/depression (17%), usual activities (12%), and self-care (1%) (**Figure 1**). While the proportions of reported problem slightly increased in mobility, anxiety/depression, usual activities and slightly decreased in pain/discomfort and self-care, no changes in any of the five individual items were statistically or clinically significant (**Table 2**, **Figure 2**).

**Figure 3:**
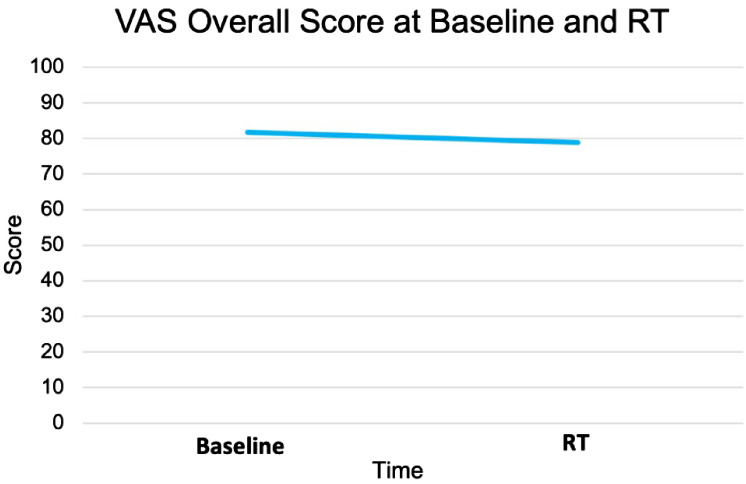
EQ VAS overall score at baseline and at SBRT initiation.

**Figure 4:**
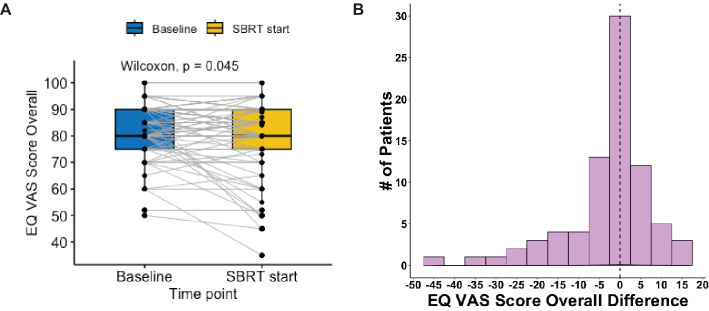
Distribution of EQ VAS score overall pre- vs. post-relugolix therapy. **(A)** Paired boxplot of EQ-VAS score overall, in which each box has horizontal lines indicating median values and extends from the 25th to the 75th percentile and in which each gray line connecting two boxes indicates individual patient. **(B)** Histogram of EQ-VAS score overall difference (start–baseline) and the vertical line at 0 indicates patient median.

The EQ VAS score was statistically significantly higher at baseline (mean ± SD: 82 ± 10) than the paired score at the start of SBRT (79 ± 14, p = 0.02) (**Table 2**, **Figure 3**). This change was not clinically significant as evaluated by MID. The paired difference in VAS score overall between the two time points had a median of 0 (IQR: −5, 2.5) (**Figure 4**). Patients were divided into two groups, one group whose VAS score overall remained the same or increased (62%) versus the other whose score decreased (38%). These two groups had no statistically significant differences across demographic and clinical characteristics, as reported in **Table 1**.

## 4 Discussion

Growing evidence suggests that achieving profound castration (serum testosterone levels of <20 ng/dl) may yield better outcomes in specific clinical contexts, although current guidelines define effective castration as testosterone levels of <50 ng/dl (13). While the impact of testosterone levels of <20 ng/dl on HRQoL remains ambiguous, it is reassuring to note that 87% of our patient cohort achieved profound castration without any clinically meaningful deterioration in HRQoL. This result aligns with findings from the Radiation Therapy Oncology Group (RTOG) 0815 randomized trial, which investigated dose-escalated radiation with or without short-term androgen suppression in intermediate-risk prostate adenocarcinoma (5). In that study, short-term androgen suppression using injectable GnRH receptor agonists combined with oral antiandrogen therapy over a 6-month period demonstrated no effect on EQ-5D VAS scores up to 5 years following radiation therapy. These findings are particularly relevant for intermediate-risk patients considering the addition of relugolix to their SBRT regimen.

Similar to other SBRT cohorts, our patient population was older and presented with moderate reductions in baseline HRQoL before treatment. While androgen deprivation therapy is associated with musculoskeletal pain (30%) and fatigue (25%) (8), we did not see increased difficulties with self-care, usual activities, mobility, or pain/discomfort in patients receiving short-term relugolix. Notably, 41% of our study population was non-white, representing diverse socioeconomic and racial backgrounds (14). The absence of adverse HRQoL impacts in this socially diverse cohort further supports the safety of short-term relugolix use in these patients.

We also conducted a deeper investigation into a subset of eight patients who had “severely” negative effect from ADT defined as those whose EQ VAS score decreased by 20 or greater. An arbitrary cutoff point of 20 was decided because there were recent studies, which suggested more flexible criteria to define the clinical significance in differences as two or three times the conventional MID (0.5 of SD at baseline) (15) or as the standard error of the measure (SEM) represented by $SD*\sqrt{1-Cronbach^{\prime }s\;alpha}$ (16).

Interestingly, these eight patients had no significant differences in all of the reported baseline demographic and clinical characteristics compared to the rest of the cohort. Among these eight patients, the majority reported to have “some problems” from “no problem” in baseline in the mobility (n = 4) and usual activities (n = 4) sections (**Table 3**).

**Table 3:** Patients whose EQ VAS score decreased by 20 points or greater (n = 8).

Pt #	VAS score difference	Baseline	Start of RT	Changed EQ-5D Sections
1	−45	11111	11111	None
2	−35	11111	21221	Mobility, usual activities, pain/discomfort
3	−30	11121	21222	Mobility, usual activities, anxiety/depression
4	−25	11111	11111	None
5	−25	11122	21122	Mobility
6	−20	11122	21222	Mobility, usual activities
7	−20	11112	11212	Usual activities
8	−20	11111	11111	None

Also, the EQ VAS score decrease did not seem to reflect on the individual items, as three of these eight patients reported to have no change in all five sections and remained as good health state (“11111”), including the patient who reported to have the greatest decrease in the EQ VAS score of 45 points. This could suggest that these five dimensions may not have adequately represent the side effects the patients experienced from the hormonal therapy as represented in the EQ VAS score.

One possible missing component accounting for the observed discrepancy is patient-reported fatigue, a commonly reported bothersome effect of hormonal therapy; our previous study showed that while most patients had significantly increased patient-reported fatigue, they were not impacted in the self-care aspect (17). It is also worth noting that a recent study conducted by our team on the effects of relugolix on patient-reported sexual function found that, while neoadjuvant relugolix was linked to a significant decline in sexual function, the majority of patients did not express concern about this effect (18) Last, the major advantage of relugolix, particularly for patients experiencing significant side effects from hormonal therapy, is that the medication effects can be resolved quickly when stopped (8).

### 4.1 Strengths and limitations

The rigorous standard in overall survey methodology and quality of data collection were assured by experienced clinical research data managers at an identical hospital to that where the patients received clinical treatment. Patients in our cohort were treated in one clinical site, which ensures less heterogeneity of treatment. Although patient reported, this study’s outcomes are based on EQ-5D, a widely used and respected HRQoL survey globally (19). Selecting a generic measure, such as the EQ-5D, as the main outcome allowed this study to encompass a spectrum of HRQoL that might be overlooked in measures focused solely on specific physical, functional, or mental health facets.

The external validity of our investigation is constrained by the small number of participants and slight differences in the treatment schedule. Although we intended for all patients to receive relugolix for 4–6 months starting 2 months prior to SBRT, there were variations in the timing of therapy. We did not explore how scheduling divergences may have affected outcomes.

## 5 Conclusions

Future studies should focus on comparing it to GnRH agonist-induced change in quality of life. Neoadjuvant relugolix administered before prostate SBRT did not have a clinically meaningful impact on patient-reported health-related quality of life. There were no statistically significant reductions in any of the five individual items comprising EQ-5D-3L. Additional research should be directed toward comparing quality-of-life outcomes for patients on relugolix to those resulting from GnRH agonist treatment.

## Data Availability

The raw data supporting the conclusions of this article will be made available by the authors, without undue reservation.
